# Acute abdomen due to late retroperitoneal extravasation from a femoral venous catheter in a newborn

**DOI:** 10.1590/S1516-31802002000200007

**Published:** 2002-03-02

**Authors:** Jaques Sztajnbok, Eduardo Juan Troster

**Keywords:** Acute abdomen, Femoral venous, catheterization, Extravasation, Parenteral nutrition, Newborn, Abdome agudo, Cateterismo venoso femoral, Extravasamento, Hipe- ralimentação parenteral, Recém-nascido

## Abstract

**CONTEXT:**

The use of parenteral nutrition via a central venous catheter is a common practice in the neonatal intensive care setting. Extravasation of the infusate leading to an acute abdomen is a complication that has only rarely been documented. This report describes the case of a premature infant with a femoral catheter placed in the inferior vena cava, who developed an acute abdomen as a result of late retroperitoneal extravasation of parenteral nutrition.

**CASE REPORT:**

A pre-term infant receiving total parenteral nutrition via a femoral venous catheter developed an acute abdomen five days after the catheter placement. Extravascular catheter migration to the retroperitoneal space and extravasation of the infusate was diagnosed by contrast injection. Withdrawal of the catheter was followed by prompt cessation of the signs and full recovery from the acute abdomen, without the need for surgery. A review of the literature is presented, emphasizing the clinical and therapeutic aspects of this unusual complication from femoral venous catheterization and parenteral nutrition.

## RESUMO

**CONTEXTO:**

A utilização de cateter femoral para nutrição parenteral é uma prática comum em terapia intensiva pediátrica com algumas complicações relatadas. Uma dessas complicações raramente descritas é a migra- ção tardia do cateter femoral levando a extra- vasamento retroperitoneal da solução infundida, situação essa caracteristicamente acompanhada de quadro clínico e laboratorial de abdome agudo.

**RELATO DE CASO:**

Recém-nascido pré-termo recebendo nutrição parenteral total por cateter femoral apresentou quadro de abdome agudo no quinto dia após a introdução do cateter. A migração extravascular da ponta do cateter com extravasamento retroperitoneal da solução foi diagnosticada por estudo contrastado. A simples retirada do cateter foi suficiente para a melhora do quadro clínico de abdome agudo, sem necessidade de cirur- gia. Os autores apresentam uma revisão de literatura com ênfase nos aspectos clínicos, diagnósticos e terapêuticos desta rara com- plicação tardia de cateterização venosa femoral e nutrição parenteral em crianças.

## INTRODUCTION

The use of parenteral nutrition via a central venous catheter has become a common practice in the neonatal intensive care setting. Some complications due to this procedure are well known, but extravasation of the infusate leading to an acute abdomen has only rarely been documented. This report describes the case of a premature infant with a femoral catheter placed in the inferior vena cava, who developed an acute abdomen as a result of late retroperitoneal extravasation of parenteral nutrition.

## CASE

A 1380-gram premature male newborn, born at the 34^th^ week of gestation, was admitted to the pediatric intensive care unit with a diagnosis of hyaline membrane disease. This required exogenous surfactant therapy and mechanical ventilatory support.

On the 11^th^ day of life, he underwent partial colectomy because of necrotizing enterocolitis and the use of vancomycin plus imipenem was started. A right femoral vein catheter (22GA, Becton Dickinson, Sandy, Utah, USA) was inserted by cut-down, with the tip placed below the renal veins in the inferior vena cava, and parenteral nutrition was instituted. The infant presented a good recovery after surgery. Bowel sounds were audible on the 2^nd^ postoperative day and colostomy became functional on the third postoperative day.

On the fifth postoperative day, however, the infant suddenly deteriorated. The abdomen became distended with diffuse tenderness and induration of the anterior abdominal wall. Bowel sounds were absent. The child had a temperature of 38.2 °C and an increased leukocyte count of 32,200/mm^3^ (2 metamyelocytes, 8 band forms, 71 segmented neutrophils, 12 lymphocytes, 4 eosinophils, and 3 monocytes), with a platelet count of 159,000/mm^3^.

**Figure 1:**
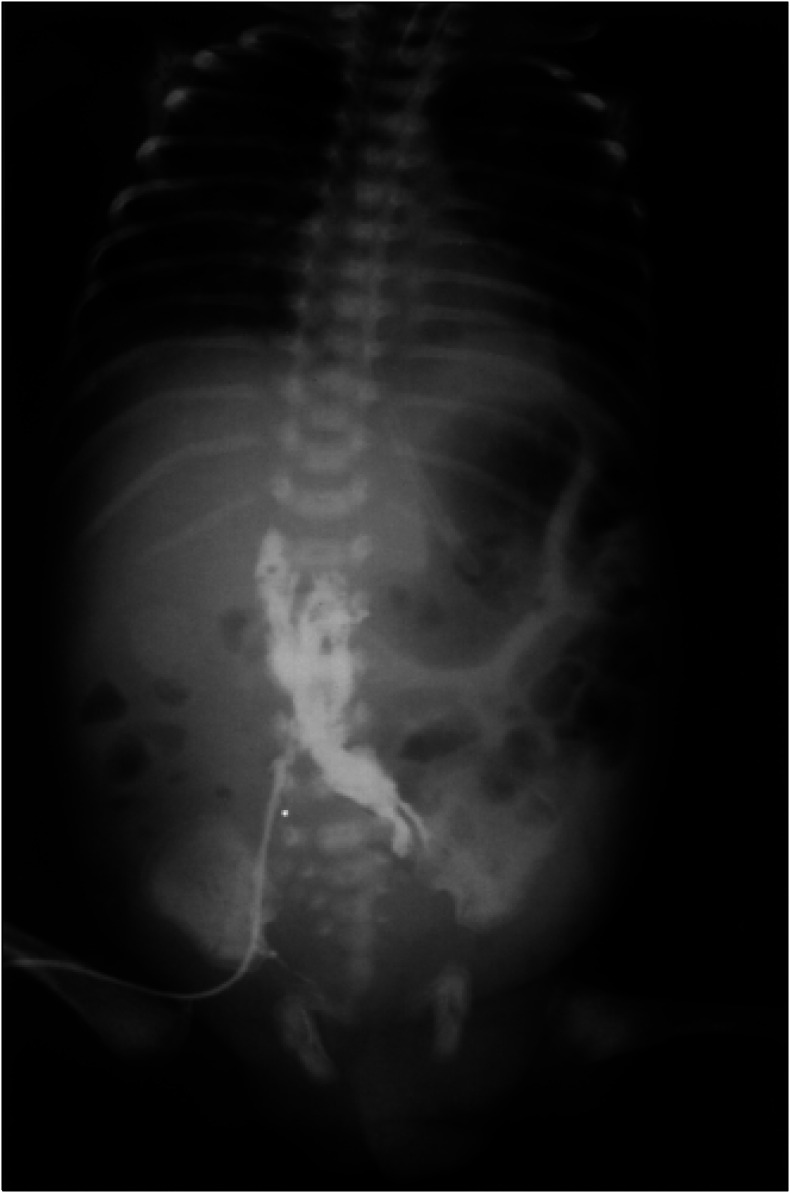
Right femoral vein catheter. Contrast injection demonstrates retroperitoneal extravasation that occurred five days after the catheter placement.

The femoral catheter was aspirated without blood return and a contrast that was then injected through this catheter revealed retroperitoneal extravasation of the dye (Figure 1). The right femoral catheter was removed and another one was then inserted into the left femoral vein via a distinct cut-down, so that parenteral nutrition could be maintained.

Within 24 hours of the catheter removal, the infant had a soft abdomen and the return of gastrointestinal function. Both the abdominal tenderness and the marked induration of the abdominal wall were completely resolved. The new catheter was well tolerated without any complication until its removal on the 10^th^ day after its insertion.

## DISCUSSION

Establishing central venous access is critical in the sick neonate. Patients in neonatal intensive care units require parenteral antibiotics for the treatment of infections. They need total parenteral nutrition when they cannot take in adequate nutrition by enteral means and parenteral access for lifesupport drugs. In addition, neonates experience large fluid losses because of their relatively large body surface area, and require reliable access for hydration.^1^

The placement of peripheral intravenous lines (PIV) can provide the majority of the fluid and nutritional needs of the neonate. The primary problem with peripheral venous catheters is the difficulty in placing and maintaining them. There are also several complications such as phlebitis and extravasation that lead to soft tissue necrosis and infection, usually requiring frequent changes of the lines.^2^

Peripherally inserted central venous catheters are more reliable than PIV lines. Factors indicating their placement include the need for access lasting longer than 3 days or a lack of alternative sites. Their advantages over PIVs include increased caloric administration, decreased venipuncture and ease of maintenance. The rate of insertion complications is reduced in relation to standard venous lines.^1^ Such complications include mechanical problems, dislodgment, venous thrombosis and infection.^1^

Central venous lines are the last option when peripheral access is necessary. Placement is either by means of percutaneous techniques or cut-down. The most common sites are the internal jugular, subclavian, femoral, saphenous, facial and external jugular veins. Puncture of the subclavian or internal jugular veins is more difficult and is associated with an increased risk of pneumothorax. A groin line is easier to place because the patient can be restrained better and pneumothorax is not a concern there. The saphenous vein is preferred because there is a decreased risk of venous hypertension in the leg. The risk of infection may be higher with groin lines, but this issue remains controversial.^1^

The most commonly reported complications include venous thrombosis and edema of the distal lower extremities.^3^ Extravasation from a femoral venous catheter is an exceedingly rare event, with only a few cases described to date.^4-9^ It appears that localized chemical phlebitis induced by the high osmolarity of the infusate, followed by subsequent catheter erosion, is the most likely cause of this complication.^8^ It has been suggested that previous or concomitant staphylococcal infection may play a role in this occurrence.^7^

Extravasation has been reported as occurring both intraperitoneally and retro- peritoneally.^4-9^ Interestingly, regardless the extravasation site, most of these cases presented with acute abdomen and shared common findings. These findings included abdominal tenderness and distention,^4-9^ fever,^4,6,7,9^ leukocytosis,^4,6,7,9^ a characteristic induration of the abdominal wall secondary to infiltration of its soft tissues,^4-7^ failure of the catheter to provide an adequate blood reflux when aspirated,^4-9^ and the fact that, in almost all of the cases, the catheters were being used for parenteral nutrition support.^5,7-9^ In most of these cases, the diagnosis of extravasation was ultimately confirmed by contrast injection into the catheter or by peritoneal paracentesis. All cases presented prompt recovery from both the laboratory and clinical changes, immediately after the cessation of infusions and the removal of the catheters.^4-9^ These findings indicate that the problems of femoral venous catheter extravasation during parenteral nutrition are self-correcting after the removal of the catheter, and that this removal is usually sufficient therapy.

Physicians need to be aware of such conditions when faced with a child showing signs and symptoms of acute abdomen. If the child has a femoral catheter, the possibility of catheter extravasation should be considered. Its position should be checked by contrast injection and the catheter should be aspirated. If the catheter is failing to provide adequate blood reflux or the x-ray evidence shows extravasation of the dye, the catheter should then be removed. This would usually be sufficient therapy in itself. Catheter extravasation should also be included in the differential diagnosis of acute abdomen in infants or children who have femoral catheters positioned in the inferior vena cava. Its early recognition could prevent unnecessary laparotomy, thereby considerably reducing the risks for the patient.
